# The evidence base for circulating tumour DNA blood-based biomarkers for the early detection of cancer: a systematic mapping review

**DOI:** 10.1186/s12885-017-3693-7

**Published:** 2017-10-23

**Authors:** Ian A. Cree, Lesley Uttley, Helen Buckley Woods, Hugh Kikuchi, Anne Reiman, Susan Harnan, Becky L. Whiteman, Sian Taylor Philips, Michael Messenger, Angela Cox, Dawn Teare, Orla Sheils, Jacqui Shaw, For the UK Early Cancer Detection Consortium

**Affiliations:** 1 WHO Classification of Tumours Group, International Agency for Research on Cancer (IARC), World Health Organization, 150 Cours Albert Thomas, 69372 Lyon, CEDEX 08 France; 2 Faculty of Health and Life Sciences, Coventry University, Priory Street, Coventry, CV1 5FB UK; 3 Institute of Ophthalmology, University College London, EC1V 9EL, London, UK; 4 The School of Health and Related Research, The University of Sheffield, Regent Court, 30 Regent Street, Sheffield, S1 4DA UK; 5 Department of Pathology, University Hospitals Coventry and Warwickshire, Coventry, CV2 2DX UK; 6 London North West Healthcare NHS Trust, Northwick Park Hospital, Watford Road, Harrow, HA1 3UJ UK; 7 Warwick Medical School, University of Warwick, Coventry, CV4 7AL UK; 8 Leeds Centre for Personalised Medicine and Health, University of Leeds and NIHR Diagnostic Evidence Co-Operative Leeds, Leeds Teaching Hospitals NHS Trust, Leeds, LS9 7TF UK; 9 Sheffield Institute for Nucleic Acids, Department of Oncology and Metabolism, The University of Sheffield, Medical School, Beech Hill Road, Sheffield, S10 2RX UK; 10 Sir Patrick Dun Research Laboratory, Central Pathology Laboratory, St James’s Hospital & Trinity College Dublin, Dublin 8, Ireland; 11 University of Leicester, Robert Kilpatrick Clinical Sciences Building, Leicester Royal Infirmary, Leicester, LE2 7LX UK

**Keywords:** cfDNA, ctDNA, Cancer, Detection, Diagnosis, Liquid biopsy

## Abstract

**Background:**

The presence of circulating cell-free DNA from tumours in blood (ctDNA) is of major importance to those interested in early cancer detection, as well as to those wishing to monitor tumour progression or diagnose the presence of activating mutations to guide treatment. In 2014, the UK Early Cancer Detection Consortium undertook a systematic mapping review of the literature to identify blood-based biomarkers with potential for the development of a non-invasive blood test for cancer screening, and which identified this as a major area of interest. This review builds on the mapping review to expand the ctDNA dataset to examine the best options for the detection of multiple cancer types.

**Methods:**

The original mapping review was based on comprehensive searches of the electronic databases Medline, Embase, CINAHL, the Cochrane library, and Biosis to obtain relevant literature on blood-based biomarkers for cancer detection in humans (PROSPERO no. CRD42014010827). The abstracts for each paper were reviewed to determine whether validation data were reported, and then examined in full. Publications concentrating on monitoring of disease burden or mutations were excluded.

**Results:**

The search identified 94 ctDNA studies meeting the criteria for review. All but 5 studies examined one cancer type, with breast, colorectal and lung cancers representing 60% of studies. The size and design of the studies varied widely. Controls were included in 77% of publications. The largest study included 640 patients, but the median study size was 65 cases and 35 controls, and the bulk of studies (71%) included less than 100 patients. Studies either estimated cfDNA levels non-specifically or tested for cancer-specific mutations or methylation changes (the majority using PCR-based methods).

**Conclusion:**

We have systematically reviewed ctDNA blood biomarkers for the early detection of cancer. Pre-analytical, analytical, and post-analytical considerations were identified which need to be addressed before such biomarkers enter clinical practice. The value of small studies with no comparison between methods, or even the inclusion of controls is highly questionable, and larger validation studies will be required before such methods can be considered for early cancer detection.

## Background

The early detection of cancers before they metastasise to other organs allows definitive local treatment, resulting in excellent survival rates. This is particularly true for breast cancer, but also others, including lung and colorectal cancer [1]. Early detection and diagnosis has therefore been a major goal of cancer research for many years, and the concept of early detection from a blood sample has been the focus of considerable effort. However, to date no blood biomarkers have had sufficient sensitivity and specificity to warrant their clinical use for early cancer detection, and their potential remains unrealised [2]. Hanahan and Weinberg [3] identified the major biological attributes of cancer, and it is apparent that most if not all of these biological processes give rise to biomarkers present in blood [4]. Circulating cell free DNA produced from cancers is known as circulating tumour DNA (ctDNA), and represents a subset of the circulating DNA (cfDNA) normally present at low levels in the blood of healthy individuals.

Since the first description of circulating cfDNA in blood [5, 6], it has become clear that total ctDNA levels rise in a number of disorders in addition to cancer including myocardial infarction [7], serious infections, and inflammatory conditions [8], as well as pregnancy where it can be used for prenatal diagnosis [9]. The source of this DNA appears to be mainly the result of cell death – either by necrosis or apoptosis [5, 9–11]. A raised ctDNA level is therefore non-specific, but may indicate the presence of serious disease. In blood, ctDNA is always present as small fragments, which makes assay design challenging [12]. Nevertheless, many analytical methods are available to measure ctDNA, and the field is rapidly maturing to the point where it may be clinically relevant to many patients.

In 2014, the UK Early Cancer Detection Consortium (ECDC) conducted a rapid mapping review of blood biomarkers of potential interest for cancer screening [13], and identified 814 biomarkers, including 39 ctDNA biomarkers. This paper uses the list generated from the mapping review, updated with relevant publications published since its completion to discuss the candidacy of ctDNA markers for early detection of cancer.

## Methods

Our mapping review [13] conducted comprehensive searches of the electronic databases Medline, Embase, CINAHL, the Cochrane library, and Biosis to obtain relevant literature on blood-based biomarkers for cancer detection in humans (PROSPERO no. CRD42014010827). The search period finished in July 2014, therefore the searches have been updated to December 2016 using the same search terms. The abstracts of the publications retrieved were reviewed to identify those with validation data (usually indicated by case-control design) and to determine what ctDNA biomarkers had been measured in serum or plasma. Full details of the methods used are published elsewhere [13], and described briefly here. English language publications of any sample size were eligible and the full eligibility criteria used are provided in Table 1.

**Table 1:** Search criteria for ctDNA publications

Inclusion Criteria	Exclusion Criteria
English language studies	Studies published in non-English language
Studies within last seven years (2010–2016)	Studies published in 2009 or earlier
Controlled studies	Citation titles without abstracts
Validation Studies (comparison with controls)	Parallel publications and reviews based on the same or overlapping patient populations^a^
Cancer detection/ diagnosis/screening	Prognosis or prediction (treatment response) associated markers
Biomarkers measured in blood plasma or serum (markers or biomarkers)	Tissue, blood cells, or other bodily fluid samples
DNA (including cfDNA and ctDNA)	Abstracts of panels which do not state which biomarkers are studied
Human DNA	Viral and microbial DNA

^a^Reviews and meta-analyses are cited, but not considered as evidence, but studies were included if they appeared to contain new data

The search strategy was deliberately inclusive, using keywords and subject headings as follows, to provide a comprehensive list of those ctDNA candidate biomarkers that had been used to identify cancers from blood samples. The search terms included ‘cancer’ ‘diagnosis’, ‘markers’, ‘blood’, and ‘screening’ with ‘DNA’, ‘cfDNA’, or ‘ctDNA’. Keywords and subject headings were determined by members of the ECDC working with the review team at the University of Sheffield. The results of the searches were collated in an Endnote database and results tabulated, with references, size of study, and methods used. To avoid bias, two reviewers conducted screening; references identified by either as relevant were included for further inspection. Those featuring ctDNA with data related to diagnosis or detection of three or more types of cancer were identified and retained for closer scrutiny to determine their potential utility.

## Results

**Fig. 1:**
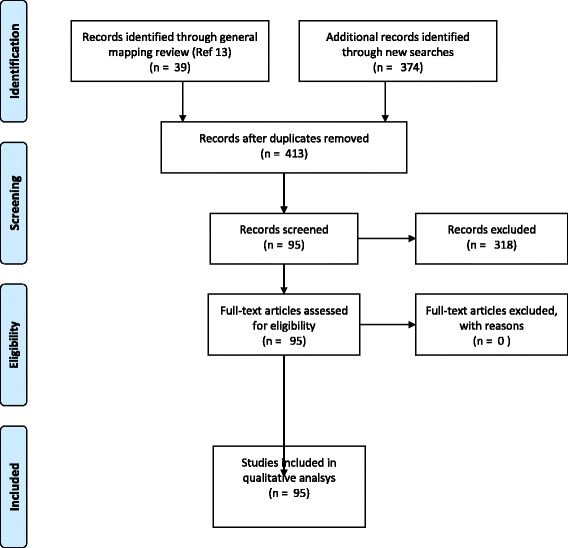
PRISMA diagram

Following the updated searches and study selection, a total of 84 ctDNA markers were identified from 94 individual publications (Table 2 and Fig. 1).

**Table 2:** Individually identified markers with detection ability in ctDNA

No	Biomarker	Acronym	Cancer	DNA alteration	Assay type (qPCR, ddPCR, BEAMing, NGS, Other)	Size Cases (controls)	Plasma or Serum	Refs
1	14–3-3 sigma	14–3-3 s	Breast	Methylation	qPCR	106 (74)	Serum	[48]
2	absent in melanoma 1	AIM1; Beta/gamma crystallin domain-containing protein 1	Lung	Methylation	qPCR	76 (30)	Serum	[62]
3	ADAM: metallopeptidase with thrombospondin type 1 motif, 1	ADAMTS1	Pancreatic	Methylation	qPCR	42	Serum	[63]
4	Adenomatous Polyposis Coli	APC	Lung	Methylation	qPCR	76 (30)	Serum	[62]
			CRC	Mutation	qPCR	33 (10)	Plasma	[64]
			Testicular	Methylation	qPCR	73 (35)	Serum	[47]
			CRC	Mutation	qPCR	191	Plasma	[65]
			CRC	Methylation	qPCR	33	Serum	[53]
			CRC	Mutation	PCR	104	Serum	[66]
			Ovarian	Methylation	qPCR	87 (62)	Serum	[67]
			Renal	Methylation	PCR	35 (54)	Serum	[68]
			Breast	Methylation	qPCR	36 (30)	Plasma	[69]
			Lung	Methylation	qPCR	110 (50)	Plasma	[70]
			Renal	Methylation	qPCR	27 (15)	Plasma	[71]
			CRC	Methylation	PCR	60 (100)	Plasma	[72]
5	ALU repeat	Alu 115 bp	Breast	NA	qPCR	39 (49)	Plasma	[22]
		Alu 247 bp	Pancreatic	NA	qPCR	73 (43)	Plasma	[73]
			CRC	NA	qPCR	50 (35)	Plasma	[20]
			Breast	NA	qPCR	293 (100)	Plasma	[19]
			Thyroid	NA	qPCR	176 (19)	Plasma	[24]
			CRC	NA	qPCR	104 (173)	Serum	[23]
6	basonuclin 1	BNC1	Pancreatic	Methylation	qPCR	42	Serum	[63]
7	BIN1	BIN1	Breast	Methylation	qPCR	76 (30)	Serum	[62]
8	BLU	BLU	Lung	Methylation	qPCR	63 (36)	Plasma	[74]
9	BRAF	BRAF (V600E)	Melanoma	Mutation	qPCR	221	Both	[17]
			Lung	Mutation	NGS	68 (107)	Plasma	[75]
			LCH	Mutation	qPCR	30	Plasma	[76]
			CRC	Mutation	qPCR	106	Plasma	[77]
			Thyroid	Mutation	qPCR	77	Plasma	[78]
			CRC	Mutation	BEAMing	503	Plasma	[21]
			CRC	Mutation	qPCR	191	Plasma	[65]
10	BRCA1	BRCA1	Breast	Methylation	qPCR	89	Serum	[79]
			Breast	Methylation	qPCR	36 (30)	Plasma	[69]
			Ovarian	Methylation	PCR	50	Serum	[80]
			Ovarian	Methylation	PCR	33 (33)	Plasma	[81]
11	CALCA	CALCA	Ovarian	Methylation	PCR	30 (30)	Plasma	[82]
12	CDH1	CDH1	Ovarian	Methylation	qPCR	87 (62)	Serum	[67]
13	CDH13	CDH13	Lung	Methylation	qPCR	63 (36)	Plasma	[74]
			Lung	Methylation	qPCR	110 (50)	Plasma	[70]
14	CDO1	CDO1	Various	Methylation	qPCR	150 (60)	Plasma	[83]
15	CHD1	CHD1	Lung	Methylation	qPCR	76 (30)	Serum	[62]
16	CST6	CST6	Breast	Methylation	qPCR	196 (37)	Plasma	[84]
			Breast	Methylation	qPCR	36 (30)	Plasma	[69]
17	CHRM2	CHRM2	Gastric	Methylation	qPCR	58 (30)	Serum	[85]
18	CYCD2	CYCD2	CRC	Methylation	qPCR	30 (30)	Plasma	[86]
19	DAPK1	DAPK1	HNSCC	Methylation	PCR	40 (41)	Serum	[87]
20	DCC	DCC	Lung	Methylation	qPCR	76 (30)	Serum	[62]
21	DCLK1	DCLK1	Lung	Methylation	qPCR	65 (95)	Plasma	[88]
			Lung	Methylation	qPCR	32 (8)	Plasma	[89]
22	DKK3	DKK3	Breast	Methylation	qPCR	604 (59)	Serum	[90]
23	DLEC1	DLEC1	Lung	Methylation	qPCR	110 (50)	Plasma	[70]
			HNSCC	Methylation	PCR	40 (41)	Serum	[87]
24	DNA (NOS)	DNA	Lung	NA	qPCR v Seq	30 (26)	Plasma	[91]
			Various	No	NGS	77 (35)	Plasma	[45]
			Various	No	NGS	640	Plasma	[16]
			Lung	No	qPCR	65 (44)	Plasma	[92]
			Ovarian	No	bDNA	36 (41)	Serum	[93]
25	e-cadherin	e-cadherin	Colorectal	Methylation	PCR	60 (100)	Plasma	[72]
26	EGFR	EGFR	Lung	Mutation	NGS	68 (107)	Plasma	[75]
27	EP300	EP300	Ovarian	Methylation	PCR	30 (30)	Plasma	[82]
28	ERBB2	HER2	Lung	Mutation	NGS	68 (107)	Plasma	[75]
			Breast	Amplification	qPCR	120 (98)	Plasma	[14]
			Oesphageal	Amplification	qPCR	41 (34)	Plasma	[94]
29	ESR	ESR	Breast	Methylation	qPCR	106 (74)	Serum	[48]
			Breast	Methylation	qPCR	36 (30)	Plasma	[69]
30	FAM5C	FAM5C	Gastric	Methylation	qPCR	58 (30)	Serum	[85]
31	FHIT	FHIT	Lung	Methylation	qPCR	63 (36)	Plasma	[74]
			Renal	Methylation	qPCR	27 (15)	Plasma	[71]
32	Glyceraldehyde-3-phosphate dehydrogenase	GAPDH	Breast	NA	qPCR	200 (100)	Serum	[26]
			Breast	NA	qPCR	33 (50)	Serum	[27]
			Breast	NA	qPCR	27 (32)	Serum	[28]
			Breast	NA	qPCR	33 (32)	Serum	[29]
33	GNA11	GNA11	Uveal Melanoma	Mutation	NGS	28	Plasma	[34]
34	GNAQ	GNAQ	Uveal Melanoma	Mutation	NGS	28	Plasma	[34]
35	GPC3	GPC3	Pancreatic	Methylation	qPCR	30 (30)	Plasma	[86]
36	GSTP1	GSTP1	Breast	Methylation	qPCR	89	Serum	[79]
			Breast	Methylation	qPCR	36 (30)	Plasma	[69]
			Prostate	Methylation	PCR	12 (10)	Plasma	[95]
			Prostate	Methylation	qPCR	31 (44)	Plasma	[96]
			Testicular	Methylation	qPCR	73 (35)	Serum	[47]
			Renal	Methylation	PCR	35 (54)	Serum	[68]
			Prostate	Methylation	PCR	31 (34)	Serum	[97]
37	HIC1	HIC1	CRC	Methylation	PCR	30 (30)	Plasma	[98]
			CRC	Methylation	qPCR	30 (30)	Plasma	[86]
38	HOXA7	HOXA7	Various	Methylation	qPCR	150 (60)	Plasma	[83]
39	HOXA9	HOXA9	Various	Methylation	qPCR	150 (60)	Plasma	[83]
40	HOXD13	HOXD13	Breast	Methylation	qPCR	253 (434)	Serum	[99]
41	IgH	FR3A/VLJH	Lymphoma	Clonality	NGS	75	Plasma	[43]
42	ITIH5		Breast	Methylation	qPCR	604 (59)	Serum	[90]
43	INK4A	INK4A	HCC	Methylation	Seq	66 (43)	Plasma	[100]
44	KLK10	KLK10	Lung	Methylation	qPCR	110 (50)	Plasma	[70]
45	KRAS	KRAS	Lung	Mutation	NGS	68 (107)	Plasma	[75]
			CRC	Mutation	qPCR	52	Plasma	[101]
			CRC	Mutation	qPCR	35 (135)	Plasma	[30]
			CRC	Mutation	qPCR	229 (100)	Plasma	[102]
			CRC	Mutation	qPCR	106	Plasma	[77]
			Lung	Mutation	qPCR	82 (11)	Plasma	[103]
			CRC	Mutation	BEAMing	503	Plasma	[21]
			CRC	Mutation	qPCR	191	Plasma	[65]
			CRC	Mutation	PCR	104	Serum	[66]
46	LINE1 Repeat	LINE1 79 bp	CRC	NA	qPCR	50 (35)	Plasma	[20]
		LINE1 300 bp	CRC	NA	qPCR	503	Plasma	[21]
			Breast	NA	qPCR	293 (100)	Plasma	[19]
47	MDG1	MDG1	CRC	Methylation	PCR	30 (30)	Plasma	[98]
48	Microsatellite alterations	FHIT LoH	Lung	NA	PCR	87 (14)	Plasma	[104]
		FHIT LoH	Lung	NA	PCR	32 (10)	Serum	[105]
		LoH	Oesophageal	NA	PCR	18 (22)	Plasma	[106]
		LoH	CRC	NA	qPCR	33	Serum	[53]
		3p LoH	Lung	NA	qPCR	64	Plasma	[107]
49	mitochondrial DNA	mtDNA	Breast	NA	qPCR	60 (51)	Plasma	[108]
50	MLH1	hMLH1	Breast	Methylation	qPCR	253 (434)	Serum	[99]
51	MYC	MYC	Neuroblastoma	Amplification	ddPCR	44	Plasma	[42]
52	MYF3	MYF3	Pancreatic	Methylation	qPCR	30 (30)	Plasma	[86]
53	MYLK	MYLK	Gastric	Methylation	qPCR	58 (30)	Serum	[85]
54	O(6)-methyl-guanine-DNA methyltransferase	MGMT	Lung	Methylation	qPCR	76	Serum	[62]
			CRC	Methylation	qPCR	33	Serum	[53]
			Breast	Methylation	qPCR	89	Serum	[79]
55	OPCML	OPCML	Ovarian	Methylation	qPCR	87 (62)	Serum	[67]
56	P14 ARF tumor suppressor protein gene	P14	Testicular	Methylation	qPCR	73 (35)	Serum	[47]
			Renal	Methylation	PCR	35 (54)	Serum	[68]
57	P16 cyclin-dependent kinase inhibitor 2A	P16, CDKN2A	Testicular	Methylation	qPCR	73 (35)	Serum	[47]
			Renal	Methylation	PCR	35 (54)	Serum	[68]
			Breast	Methylation	qPCR	36 (30)	Plasma	[69]
			Lung	Methylation	qPCR	63 (36)	Plasma	[74]
			Breast	Methylation	qPCR	253 (434)	Serum	[99]
			HNSCC	Methylation	qPCR	40 (41)	Serum	[87]
58	P21	P21	Breast	Methylation	qPCR	36 (30)	Plasma	[69]
59	P53		Various	Mutation	qPCR	20 (16)	Plasma	[109]
			Various	NA	qPCR	120 (120)	Plasma	[110]
			CRC	Mutation	qPCR	191	Plasma	[65]
			CRC	Mutation	PCR	104	Serum	[66]
			SCLC	Mutation	qPCR	51 (123)	Plasma	[55]
60	PCDHGB7	PCDHGB7	Breast	Methylation	qPCR	253 (434)	Serum	[99]
61	Peptidylprolyl isomerase A	cyclophilin A, gCYC, PPIA	CRC	NA	qPCR	229 (100)	Plasma	[102]
62	PIK3CA	PIK3CA	Breast	Mutation	qPCR	76	Both	[18]
			Lung	Mutation	NGS	68 (107)	Plasma	[75]
			CRC	Mutation	BEAMing	503	Plasma	[21]
			CRC	Mutation	qPCR	191	Plasma	[65]
63	Prostaglandin-endoperoxid synthase 2	PTGS2	Renal	Methylation	PCR	35 (54)	Serum	[68]
			Testicular	Methylation	qPCR	73 (35)	Serum	[47]
64	Protocadherin 10	PCDH10	CRC	Methylation	qPCR	67	Plasma	[111]
65	Retinoid-acid-receptor-beta gene	RARbeta2	Breast	Methylation	PCR	20 (25)	Plasma	[112]
			CRC	Methylation	qPCR	33	Serum	[53]
			Renal	Methylation	PCR	35 (54)	Serum	[68]
			Lung	Methylation	qPCR	63 (36)	Plasma	[74]
66	RASSF1A	RASSF1A	Breast	Methylation	PCR	93 (76)	Plasma	[113]
			Breast	Methylation	PCR	20 (25)	Plasma	[112]
			Breast	Methylation	qPCR	39 (49)	Plasma	[22]
			Breast	Methylation	qPCR	604 (59)	Serum	[90]
			Melanoma	Methylation	qPCR	84 (68)	Plasma	[114]
			Lung	Methylation	qPCR	76 (30)	Serum	[62]
			Testicular	Methylation	qPCR	73 (35)	Serum	[47]
			CRC	Methylation	qPCR	33	Serum	[53]
			Ovarian	Methylation	qPCR	87 (62)	Serum	[67]
			Renal	Methylation	PCR	35 (54)	Serum	[68]
			Lung	Methylation	qPCR	63 (36)	Plasma	[74]
			Lung	Methylation	qPCR	110 (50)	Plasma	[70]
			HCC	Methylation	PCR	40 (20)	Serum	[115, 116]
			HCC	Methylation	PCR	50 (50)	Serum	[117]
			Renal	Methylation	PCR	27 (15)	Plasma	[71]
			Breast	Methylation	qPCR	253 (434)	Serum	[99]
			CRC	Methylation	PCR	30 (30)	Plasma	[98]
			Renal	Methylation	qPCR	157 (43)	Serum	[118]
			Ovarian	Methylation	PCR	50	Serum	[80]
			Ovarian	Methylation	PCR	30 (30)	Plasma	[82]
67	RUNX3	RUNX3	Ovarian	Methylation	PCR	87 (62)	Serum	[67]
68	Septin 9	Septin 9	CRC	Methylation	qPCR	97 (172)	Plasma	[119]
			CRC	Methylation	qPCR	378 (285)	Plasma	[120]
			CRC	Methylation	qPCR	60 (24)	Plasma	[121]
			CRC	Methylation	qPCR	55 (1457)	Plasma	[58]
			Lung	Methylation	qPCR	70 (100)	Plasma	[122]
			CRC	Methylation	qPCR	135 (341)	Plasma	[123]
			CRC	Methylation	qPCR	50 (94)	Plasma	[124]
			CRC	Methylation	qPCR	44 (444)	Plasma	[59]
69	SFN	SFN	Breast	Methylation	qPCR	253 (434)	Serum	[99]
70	SFRP5	SFRP5	Ovarian	Methylation	qPCR	87 (62)	Serum	[67]
71	SHOX2	SHOX2	Lung	Methylation	qPCR	188 (155)	Plasma	[125]
			Lung	Methylation	qPCR	118 (212	Plasma	[126]
72	SOX17	SOX17	Breast	Methylation	qPCR	114 (60)	Plasma	[127]
			Various	Methylation	qPCR	150(60)	Plasma	[83]
73	SLC26A4	SLC26A4	Thyroid	Methylation	qPCR	176 (19)	Plasma	[24]
74	SLC5A8	SLC5A8 SLC26A4	Thyroid	Methylation	qPCR	176 (19)	Plasma	[24]
75	SRBC	SRBC	Pancreatic	Methylation	qPCR	30 (30)	Plasma	[86]
76	TAC1	TAC1	Various	Methylation	qPCR	150 (60)	Plasma	[83]
77	human telomerase reverse transcriptase DNA	hTERT	CRC	NA	qPCR	35 (135)	Plasma	[30]
			HCC	NA	qPCR	70 (30)	Plasma	[31]
			HCC	NA	qPCR	60 (29)	Plasma	[32]
			HNSCC	NA	qPCR	200	Plasma	[33]
78	TFPI2	TFPI2	Ovarian	Methylation	PCR	87 (62)	Serum	[67]
79	THBD-M	THBD-M	CRC	Methylation	qPCR	107 (98)	Plasma & Serum	[128]
80	TIMP3	TIMP3	Renal	Methylation	PCR	35 (54)	Serum	[68]
			Breast	Methylation	qPCR	36 (30)	Plasma	[69]
81	TMS	TMS	Pancreatic	Methylation	qPCR	30 (30)	Plasma	[86]
82	UCHL1	UCHL1	HNSCC	Methylation	PCR	40 (41)	Serum	[87]
83	Von Hippel Lindau gene	VHL	CRC	Methylation	qPCR	30 (30)	Plasma	[86]
			Pancreatic	Methylation	qPCR	30 (30)	Plasma	[86]
			Renal	Methylation	qPCR	157 (43)	Serum	[118]
84	ZFP42	ZFP42	Various	Methylation	qPCR	150 (60)	Plasma	[83]

*CRC* colorectal cancer, *HNSCC* head and neck squamous cell carcinoma, *HCC* hepatocellular carcinoma, *LCH* Langerhans cell histocytosis, *SCLC* small cell lung cancer

The ctDNA biomarkers divided naturally into two groups:

I. those with potential specificity for neoplasia (ctDNA - usually mutations or DNA alterations such as methylation), and
II. those designed to measure DNA levels, which may not be specific to neoplasia.

**Fig. 2:**
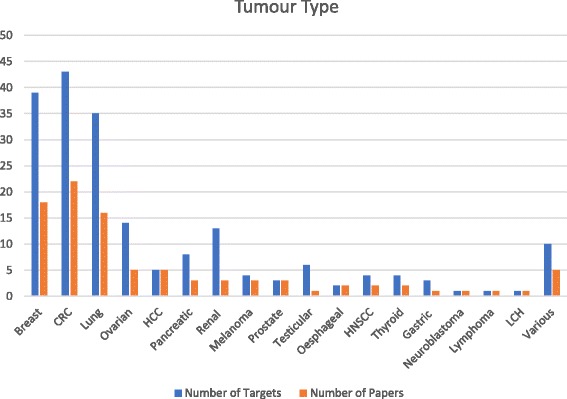
Number of targets and publications by tumour type, showing the expected concentration of studies on common cancer types. CRC, colorectal cancer; HNSCC, head and neck squamous cell carcinoma; HCC, hepatocellular carcinoma

Figure 2 shows the distribution of studies by cancer type, including two publications on amplification [12, 14], and one on clonality [15]. One of the amplification papers looked at HER2 [14], while the other examined multiple targets by NGS [12].

**Fig. 3:**
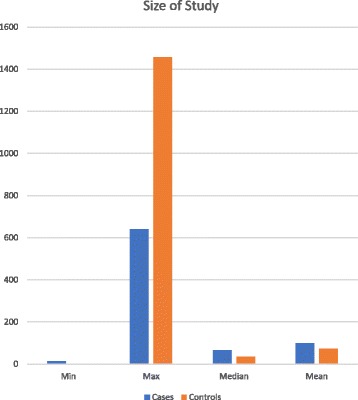
Study size. There are occasional large studies, but the vast majority are small, evidenced by the low median and averages for both cases and controls

Of the 94 publications included, 72 publications (77%) were case-control design diagnostic validation studies, and 22 were case series. The size and design of the studies varied widely. The largest study included 640 cancer patients [16]. The median study size was 65 cases, with a mean of 98 cases (range 12–640 cancer patients), indicating that the bulk of studies (67/94, 71%) included <100 patients (Fig. 3).

**Fig. 4:**
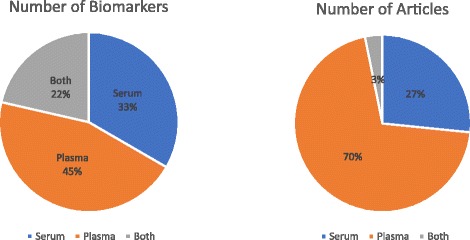
Use of serum or plasma for studies. The majority use plasma, but serum is preferred for methylation studies by some. Only three studies looked at both serum and plasma

Most publications were focussed on ctDNA in plasma (*n* = 67) rather than serum (*n* = 25) with 2 comparing both. Plasma was used for 38 markers, and serum for 28 markers, and either for 18 markers (Fig. 4). Two comparative studies of serum and plasma were conducted: one for BRAF mutations, and the other for PIK3CA mutations [17, 18].

**Fig. 5:**
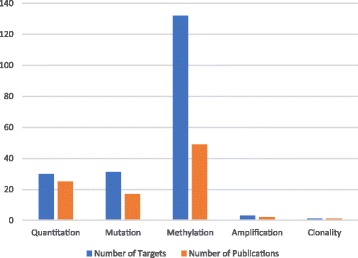
Targets: many studies looked at multiple targets, mainly either mutations or methylated genes

**Fig. 6:**
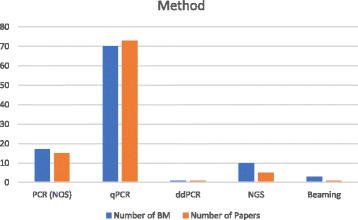
Choice of method. Most publications used just one method, but biomarkers were measurable by more than one assay in 6 instances

The target of ctDNA studies and the methods used to measure these targets varied considerably (Figs. 5 and 6 respectively). Non-specific total ctDNA levels (quantitation) were usually estimated by size distribution assays based on repeats: LINE1, and ALU were used in 3 [19–21] and 6 publications respectively [20–25]. However, some single genes were also used to measure DNA levels – particularly GAPDH in a series of 4 publications on breast cancer [26–29], and hTERT in 4 publications [30–33]. The majority of publications examined gene methylation markers (*n* = 49), though most examined methylation of multiple target genes for a particular tumour type (Fig. 5). Genes commonly mutated in cancer were also markers of interest, namely APC, BRAF, EGFR, HER2, GNAQ, GNA11, KRAS, P53, and PIK3CA. Only one gene, APC, was studied for both methylation and mutation. Few markers were used to identify particular tumour types, but some are particularly likely to occur in certain tumour types. GNAQ and GNA11 mutations have been identified in the plasma of uveal melanoma patients and are rare in other tumour types [34]. Other mutations are not tumour type-specific, and mutations in 6 of the 9 genes listed above were reported in multiple tumour types.

## Discussion

The number of publications on ctDNA is increasing rapidly [35, 36], and a recent review emphasises the potential of the field [37]. Most (71%) are small case control studies with less than 100 patients, and in our view very few studies meet the requirements of analytical validation allowing their use within accredited (ISO:15,189) clinical laboratories, though some may have unpublished commercially-held analytical validation data. The stage and size of the tumours included is variable, and few studies are large enough to give robust subgroup assessments. Larger tumours produce more ctDNA, though tumour type also has an impact [16]. The value of small studies with no comparison between methods, or even the inclusion of controls is highly questionable. Most include a statement that ‘larger studies are required’, but larger trials rarely result due to the necessary cost implications. Unless well-designed prospective studies based on sample size calculations are performed, there is little likelihood of such methods reaching clinical practice for the detection of cancer at an early stage. There is also a likelihood of bias in that negative results for these markers are rarely if ever reported, and unlike clinical trials, there is no requirement for the registration of diagnostic validation studies. The use of ctDNA for early cancer detection comes under existing molecular pathology guidance, which emphasises the requirements for careful pre-analytical preparation, analysis, and reporting of results [38]. It is important that studies adhere to the Standards for Reporting of Diagnostic Accuracy Studies (STARD) guidance [39], and regional guidance (e.g. US Food and Drug Adminstration (FDA); UK National Institute for Health and Care Excellence (NICE); Clinical & Laboratory Standards Institute (CLSI)). It is hardly surprising then that, to date, no ctDNA markers have made it into screening programmes, due in part to the economic feasibility of completing the necessary stages of validation [40]. Nevertheless, there is encouraging evidence that ctDNA can be used to detect cancers of many types [16], and the poor quality of many studies should not detract from this fact.

A plethora of methods are available for ctDNA measurement, which have been well reviewed elsewhere [41]. BEAMing, PCR clamping methods, and deep sequencing using NGS are now the most commonly used [42, 43] and are widely regarded as the most sensitive methods currently available. A recent report of copy number variation (CNV) in breast cancer is not surprising given the ability of this method to detect such changes in pregnancy [15]. However, it should be noted that many of these methods are expensive. The development of highly sensitive NGS methods for ctDNA may prove necessary to obtain the best results [44], but large blood samples (> 10 ml may be needed as the number of DNA molecules present in small samples is often low) [45]. This may be at odds with the key requirement of cost effectiveness for screening programmes, and in our view this represents a real challenge for ctDNA. The problem is probably not insuperable if automation allows the integration of such methods into large blood sciences laboratories, but this is not as yet the case.

As ctDNA is composed largely of short fragments, short amplicons are required for maximum sensitivity of PCR reactions, particularly if mutations are being detected [46]. This is compounded by DNA loss in some reactions, particularly bisulphite modification of DNA, and it may be preferable to use nuclease protection assays [47, 48]. Methylation of key genes involved in carcinogenesis can be found in ctDNA, and has been studied by many groups, but it should be noted that substantial numbers of normal controls also have methylation of ctDNA for these genes [49].

It is clear that high sensitivity methods will be needed if ctDNA is to be used for early cancer detection. Several factors affect the sensitivity of ctDNA measurement. The first is the extraction method, and there are as yet too few studies which have compared the different options available, which now include automated instruments as well as manual extraction systems [50, 51]. The proportion of tumour derived DNA (ctDNA) in total cfDNA is greater in plasma than serum, and the higher ctDNA levels in serum are due to leakage from leukocytes during clotting [17]. The dilution effect for ctDNA in serum results in a reduced ability to detect mutations, particularly by methods with low analytical sensitivity [50]. Most groups working in the field realise this, and the majority of publications now look at plasma rather than serum.

Several publications were noteworthy, including one influential study which did not include healthy controls [16]. However, the comparison of DNA levels and multiple mutations in plasma from many different tumours types is helpful [44], and makes it clear that some tumours (e.g. gliomas) do not have high ctDNA levels in plasma, as previously found when comparing CSF with plasma [52]. This is also one of several publications that examines early stage disease, and shows that patients with localised disease have lower ctDNA levels [16]. Few publications have examined the ability of ctDNA to detect smaller tumours, though all agree that ctDNA levels increase as tumours enlarge [42].

Choice of target also influences results: the use of LINE1 and ALU repeats allows quantitative size distribution of DNA to be measured. Several publications suggest that this can distinguish cancer, and even pre-cancerous conditions from controls [30]. The size distribution of CRC appears to be different from other tumours due to first pass hepatic metabolism [20, 53]. Absolute quantitation by single gene methods such as GAPDH or hTERT will result in lower estimates of DNA content, and it is likely that this is due to the higher sensitivity of the ALU and LINE1 assays [30].

The use of mutations common within cancers is attractive, and the use of ctDNA to provide companion diagnostic information in patients in whom biopsy material is not available is now entering practice [54]. However, it should be noted that such mutations in P53 can occur in the blood of healthy controls, and could give rise to substantial numbers of false positive results [55].

Septin 9 methylation is often regarded as a model for future work [56, 57], and it is notable that there are some large studies [58] within the evidence base for the use of this marker in colorectal cancer, often used in addition to other markers, such as faecal occult blood testing (FoBT) or faecal immunohistochemical testing (FIT). Pre-analytical factors have been examined for this marker [59], including diurnal variation [60]. Plasma methylation of Septin 9 is now available as a commercial test (Epi proColon 2.0; Epigenomics AG, Berlin, Germany) which has recently obtained FDA approval for colorectal cancer screening (April 2016). This is the first blood test to be approved for cancer screening, and represents an encouraging milestone.

Other methylation targets have been studied in depth and show considerable promise. These include APC for colorectal cancer, with a large number of studies (Table 2), and SHOX3, for which a recent meta-analysis suggests that it could have an important role in the diagnosis of lung cancer [61].

There is an encouraging trend towards larger, more ambitious studies, supported by the commercial sector (e.g. (https://clinicaltrials.gov/ct2/show/NCT02889978, and https://clinicaltrials.gov/ct2/show/NCT03085888). Case control studies (particular retrospective ones) can give biased results, and prospective studies in at-risk cohorts would be more useful in examining the predictive capability of these markers. Such prospective studies should include controls proven not to have cancer. The comparison of new with existing methods (e.g. tumour markers, radiology), and competing technologies, is recommended, and often required by regulators. This has cost implications for funding bodies, but is essential if the field is to progress rapidly.

## Conclusions

While ctDNA analysis may provide a viable option for the early detection of cancers, not all cancers are detectable using current methods. However, improvements in technology are rapidly overcoming some of the issues of analytical sensitivity, and it is likely that mutation and methylation analysis of ctDNA will improve specificity for the diagnosis of cancer.

## Abbreviations

14–3-3 s: 14–3-3 sigma or tyrosine 3-monooxygenase/tryptophan 5-monooxygenase activation protein theta
ADAM: metallopeptidase with thrombospondin type 1 motif, 1
AIM1: absent in melanoma 1
ALU: Alu repeat/element 9e
APC: Adenomatous Polyposis Coli
ARF: alternate reading frame
BIN1: bridging integrator 1
BLU: zinc finger MYND-type containing 10
BM: biomarker
BNC1: basonuclin 1
bp: base pair
BRAF: B-Raf proto-oncogene, serine/threonine kinase
BRCA1: breast cancer 1, DNA repair associated
BRINP3: BMP/Retinoic Acid Inducible Neural Specific 3
CALCA: calcitonin related polypeptide alpha
CDH1: cadherin 1
CDH13: cadherin 13
CDO1: cysteine dioxygenase type 1
cfDNA: circulating cell-free DNA
CHD1: chromodomain helicase DNA binding protein 1
CHRM2: cholinergic receptor muscarinic 2
CINAHL: Cumulative Index to Nursing and Allied Health Literature
CLSI: Clinical & Laboratory Standards Institute
CRC: colorectal carcinoma
CST6: cystatin 6
ctDNA: circulating tumour DNA
CYCD2: cyclin D2
DAPK1: death-associated protein kinase 1
DCC: DCC Netrin 1 receptor
DCLK1: doublecortin like kinase 1
ddPCR: digital droplet polymerase chain reaction
DKK3: Dickkopf WNT signaling pathway inhibitor 3
DLEC1: deleted in lung and esophageal cancer 1
DNA: dexoxyribonucleic acid
ECDC: UK Early Cancer Detection Consortium
EGFR: epidermal growth factor receptor (HER1)
EP300: E1A binding protein P300
ERBB2: erb-B2 receptor tyrosine kinase 2 (HER2)
ESR: estrogen receptor 1
FAM5C: BMP/retinoic acid inducible neural specific 3 (BRINP3)
FDA: US Food and Drug Adminstration
FHIT: fragile histidine triad
FIT: faecal immunohistochemical testing
FoBT: faecal occult blood testing
GAPDH: glyceraldehyde-3-phosphate dehydrogenase
gCYC: cyclophilin A
GNA11: G protein subunit alpha 11
GNAQ: G protein subunit alpha Q
GPC3: glypican 3
GSTP1: glutathione S-transferase pi 1
HCC: hepatocellular carcinoma
HER1: human epidermal growth factor receptor 1
HER2: human epidermal growth factor receptor 2
HIC1: HIC ZBTB transcriptional repressor 1
HNSCC: head and neck squamous cell carcinoma
HOXA7: Homeobox A7
HOXA9: Homeobox A9
HOXD13: Homeobox D13
hTERT: human telomerase reverse transcriptase DNA
IgH: immunoglobulin heavy locus
INK4A: cyclin dependent kinase inhibitor 2A (CDKN2A/P16)
ISO: International Standards Organization
ITIH5: inter-alpha-trypsin inhibitor heavy chain family member 5
KLK10: kallikrein related peptidase 10
KRAS: KRAS Proto-Oncogene, GTPase
LCH: Langerhans cell histocytosis
LINE1: long interspersed nuclear element 1
LoH: loss of heterozygosity
Max: maximum
MDG1: microvascular endothelial differentiation gene 1
MGMT: O(6)-methyl-guanine-DNA methyltransferase
Min: minimum
MLH1: MutL Homolog 1
mtDNA: mitochondrial DNA
MYC: MYC proto-oncogene
MYF3: myogenic differentiation 1 (MYOD1)
MYLK: myosin light chain kinase
NGS: next generation sequencing
NICE: UK National Institute for Health and Care Excellence
NOS: not otherwise specified
OPCML: opioid binding protein/cell adhesion molecule like
P14: P14 ARF tumor suppressor protein gene
P16: P16 cyclin-dependent kinase inhibitor 2A (CDKN2A)
P21: cyclin dependent kinase inhibitor 1A
P53: tumor protein P53
PCDH10: Protocadherin 10
PCDHGB7: protocadherin gamma subfamily B7
PCR: polymerase chain reaction
PIK3CA: phosphatidylinositol-4,5-bisphosphate 3-kinase catalytic subunit alpha
PPIA: Peptidylprolyl isomerase A
PTGS2: Prostaglandin-endoperoxid synthase 2
qPCR: quantitative polymerase chain reaction
RARbeta2: Retinoid-acid-receptor-beta gene
RASSF1A: Ras association domain family member 1
RUNX3: runt related transcription factor 3
SFN: Stratifin
SFRP5: secreted frizzled related protein 5
SHOX2: short stature homeobox 2
SLC26A4: solute carrier family 26 member 4
SLC5A8: solute carrier family 5 member 8
SOX17: SRY-Box 17
SRBC: serum deprivation response factor-related gene
STARD: Standards for Reporting of Diagnostic Accuracy Studies
TAC1: tachykinin precursor 1
TFPI2: tissue factor pathway inhibitor 2
THBD-M: thrombomodulin
TIMP3: tissue inhibitor of metalloproteinase 3
TMS: tumor differentially expressed protein 1
UCHL1: Ubiquitin C-Terminal Hydrolase L1
V600E: Mutation resulting in an amino acid substitution at position 600 in BRAF, from a valine (V) to a glutamic acid (E)
VHL: Von Hippel Lindau gene
ZFP42: ZFP42 Zinc Finger Protein
